# Bendamustine as first-line treatment in patients with advanced indolent non-Hodgkin lymphoma and mantle cell lymphoma in German routine clinical practice

**DOI:** 10.1007/s00277-015-2404-1

**Published:** 2015-06-28

**Authors:** M. Becker, B. Tschechne, M. Reeb, U. Schwinger, H.-R. Bruch, M. Frank, L. Straßl

**Affiliations:** Onkologische Praxis Minden-Porta, Flurweg 13, 32457 Porta Westfalica, Germany; Praxis für Hämatologie und internistische Onkologie, Lindenstraße. 77, 31535 Neustadt, Germany; Onkologische Schwerpunktpraxis, Schneiderstr. 12, 67655 Kaiserslautern, Germany; MVZ Leuschnerstr. 1, Leuschnerstr. 12, 70174 Stuttgart, Germany; Schwerpunktpraxis Bonn, Europaring 42, 53123 Bonn, Germany; iOMEDICO AG, Hanferstr. 28, 79108 Freiburg, Germany

**Keywords:** Non-Hodgkin lymphoma, Indolent, Mantle cell lymphoma, Bendamustine, First-line, Therapy

## Abstract

Bendamustine has demonstrated clinical activity and a favorable safety profile as monotherapy or in combination with rituximab in lymphoid malignancies. As interventional trials do not always reflect clinical reality, we were interested in the treatment modalities and the outcome of bendamustine-based first-line therapy in patients with advanced indolent non-Hodgkin lymphoma (NHL) and mantle cell lymphoma (MCL) in routine practice. Between April 2010 and October 2011, 324 patients were enrolled in a prospective non-interventional multicenter study. Choice of the bendamustine regimen was at the treating physician’s discretion. Effectiveness was assessed by best response. Mean age at onset of therapy was 69 years. The majority (94 %) of the patients was treated with bendamustine in combination with rituximab at a median bendamustine dose of 177 mg/m^2^ per cycle. Most often, bendamustine was administered on days 1 and 2 (87 %) at 4-week intervals over a median of 6 cycles. Two hundred eighty-one patients qualified for evaluation of response. The overall response rate was 86 % (complete response 43 %, partial response 43 %, stable disease 10 %, progressive disease 4 %). Side effects of all grades were documented for 161 of the 323 patients (50 %), most frequently affecting blood/bone marrow (35 %). Fifty-four (17 %) patients experienced side effects of grade 3 (15 %) or grade 4 (2 %), and two patients grade 5 toxicities. Bendamustine-based first-line treatment of patients with advanced indolent NHL and MCL in clinical routine practice was assessed as effective and well tolerated in our study. Response was comparable to results from interventional clinical trials.

## Introduction

Non-Hodgkin lymphoma (NHL) accounts for a relevant proportion of the global burden of cancer. In 2008, the worldwide incidence of NHL was 199,600 new cases in men and 156,300 new cases in women [1]. NHL comprises a number of heterogeneous entities. It is usually classified by the World Health Organization (WHO) classification [2]. Indolent lymphomas represent 40 % of all NHL subtypes, and follicular lymphoma being the most frequent. They are characterized by a chronic course of the disease with successive therapies upon occurrence of relapse. Mantle cell lymphoma, a subtype with a poorer prognosis, accounts for 3–10 % of NHL [2]. For a small proportion of patients with indolent lymphoma and limited stage I–II disease, there is a curative treatment option in contrast to patients with advanced stage disease. Chemoimmunotherapy is the treatment of choice in previously untreated patients with advanced indolent and mantle cell lymphoma [3–5].

Bendamustine is an alkylating agent with a unique chemical structure containing a benzimidazole ring combining alkylating and purine analogue-like properties. It shows only partial cross resistance with other alkylating agents in vitro [6, 7]. Bendamustine has shown clinical activity as a monotherapy or in combination with rituximab in patients with relapsed or refractory lymphoid malignancies [8–12]. Of note, there has been a longstanding experience with bendamustine in Germany where the drug has been approved and used for various authorized indications. Bendamustine has become a standard of care for the treatment of indolent lymphoma. Guidelines of the National Comprehensive Cancer Network (NCCN) and the European Society of Clinical Oncology (ESMO) have adopted bendamustine as a chemotherapy standard alternative to regimens like CHOP or CVP to combine with rituximab for the treatment of advanced indolent NHL and mantle cell lymphoma [3–5].

Just recently, a randomized phase III trial in 549 patients with newly diagnosed stages III or IV indolent or mantle cell lymphoma demonstrated a significantly longer median progression-free survival (PFS) with bendamustine (90 mg/m^2^ on days 1 and 2, q4w) plus rituximab compared to R-CHOP (69.5 months vs. 31.2 months; hazard ratio 0.58, 95 % confidence interval (CI) 0.44–0.74; *p* < 0.0001). Overall response rate (ORR) did not differ between the two treatment groups and was 93 % for bendamustine and rituximab. The combination of bendamustine plus rituximab was significantly better tolerated than R-CHOP [13].

It is a commonplace that prospectively randomized, controlled trials provide the highest level of evidence for comparisons of alternative types of treatment and for defining standards of care. Yet, it is also undisputed that clinical trials are prone to selection and do not always reflect clinical reality. We explored the effectiveness and safety as well as treatment modalities of bendamustine therapy in the actual “real-life” clinical practice of first-line treatment of indolent NHL and mantle cell lymphoma.

## Patients and methods

### Study design and objectives

This prospective, non-interventional, multicenter study was performed to document the routine use of bendamustine in the first-line treatment of patients with advanced indolent non-Hodgkin lymphoma as per WHO criteria [2] to get further insight into therapy, effectiveness, and safety of bendamustine in actual clinical practice. Treatment regimens, dosing, and treatment modifications were recorded. Effectiveness was determined by documentation of best response which was assessed by the investigator as per local standard. Side effects of bendamustine were assessed according to the National Cancer Institute (NCI) Common Terminology Criteria for Adverse Events (CTCAE) version 3.0. and comorbidity by the Charlson comorbidity score [14].

The study was approved by an appropriate ethics committee. All patients had to provide signed informed consent before enrollment.

### Patient population

Patients were eligible if they had a diagnosis of advanced indolent NHL (excluding chronic lymphocytic leukemia (CLL)) or mantle cell lymphoma according to WHO classification [2] with indication for systemic therapy and the decision for a bendamustine-based therapy before enrollment. There had to be no prior treatment with chemotherapy, interferon, or rituximab. Other main exclusion criteria were severe liver damage, icterus, persistent severe myelosuppression or abnormalities of peripheral blood count, major surgery within 30 days before treatment, infections, uncontrolled cardiac disease, and pregnancy or lactation.

### Treatment

It was at the treating physician’s discretion which bendamustine-based regimen to choose for the individual patient in compliance with the former national marketing authorisation of bendamustine [15].

### Documentation and statistical analysis

Data on patient characteristics, history and prior treatment of lymphoma, lymphoma characteristics, treatment with bendamustine, response, side effects of bendamustine, diagnostic procedures, hospitalizations, and further therapies planned were documented in an electronic case report form. Treatment was documented until progression or the end of therapy with bendamustine, whatever occurred first. The scheduled observation period was 6 months. Patients were followed for further 12 weeks to continue assessment of side effects.

All efforts were undertaken to achieve a complete documentation status, covering the results that had been actually investigated. However, the non-interventional character did not allow further influence. Complete documentation status was finally obtained for 95 % of patients.

The observation period of our non-interventional study was stopped in December 2011 due to a change in marketing authorization [7, 15]. Statistical analysis was descriptive. Mean values, standard deviations, median, and minimum and maximum values are reported for continuous variables. Categorical variables are reported as absolute numbers and as relative proportions. ORR and disease control rate (DCR) are reported with 95 % CI. The effects of potentially confounding variables were estimated by logistic regression models.

## Results

### Analysis sets

**Fig. 1:**
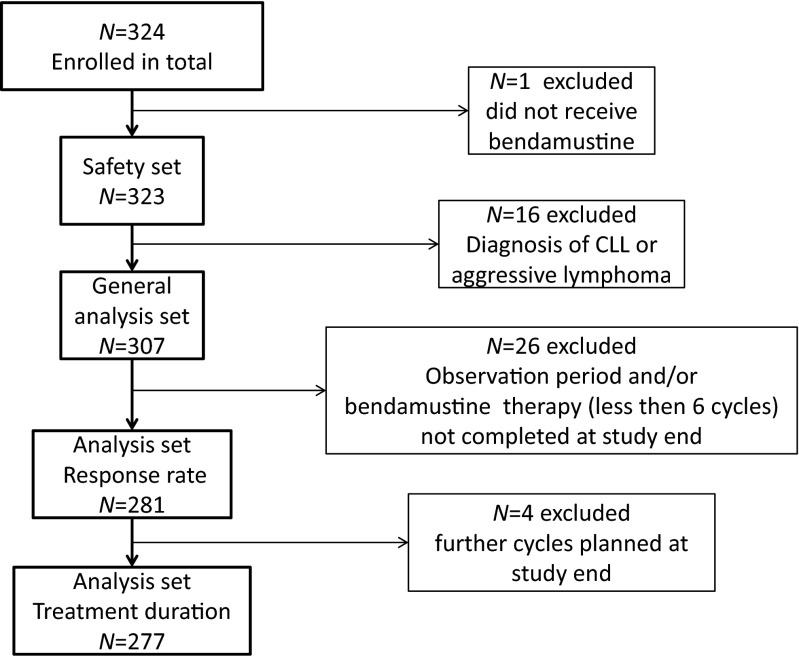
Trial profile

Between April 2010 and October 2011, 324 patients were enrolled in 57 German centers. Patients who had received at least one dose of bendamustine were included in the safety analysis set (*n* = 323). For the general analysis set (*n* = 307), 16 additional patients with a diagnosis of CLL or aggressive lymphoma were excluded. Different analysis sets were necessary for response rate (*n* = 281) and treatment duration (*n* = 277), respectively, as the observation period and/or bendamustine therapy for some patients was not completed at the end of the study on 31 December 2011 (Fig. 1).

### Patient and tumor characteristics

Complete patient and tumor characteristics of all patients from the general analysis set are listed in Table 1. Details on prognostic information are displayed in Table 2. Histology of follicular lymphoma (FL) was most common (50 %), followed by marginal zone lymphoma (MZL, 17 % incl. MALT lymphoma), immunocytoma (IC, 15 %), and MCL (12 %). Mean age at onset of therapy was 68.6 years. Patients with FL were younger than patients with other lymphoma entities (mean age 65.3 vs. 71.9 years, *p* < 0.0001). In contrast, 78 % of patients with MCL were older than 70 years (mean age: 74.2 years). The Charlson score was lower in patients with FL vs. non-FL histology (mean score 0.38 vs. 0.8, *p* = 0.0003) and higher in patients receiving bendamustine monotherapy vs. combination therapy (mean score 1.57 vs. 0.55, *p* = 0.0003). The patients presented with a mean body mass index of 26 (median: 25).

**Table 1:** Patient and tumor characteristics by lymphoma entity

Characteristic		All *N* = 307 (100 %)	Follicular lymphoma *N* = 154 (50 %)	Marginal zone lymphoma *N* = 52 (17 %)	Immunocytoma *N* = 47 (15 %)	Mantle cell lymphoma *N* = 36 (12 %)	Other entities^a^ *N* = 18 (6 %)
Age	Mean ± SD	69 ± 12	65 ± 12	70 ± 11	72 ± 9	74 ± 12	73 ± 11
	Median	71	68	71	71	76	75
	Range	27–92	37–87	49–92	52–88	27–88	50–86
Charlson score	Median	0	0	0	0	0	1.5
		*N* (%)	*N* (%)	*N* (%)	*N* (%)	*N* (%)	*N* (%)
Age groups	<65 years	106 (35)	69 (45)	18 (35)	10 (21)	5 (14)	4 (22)
	≥65 years	201 (66)	85 (55)	34 (65)	37 (79)	31 (86)	14 (78)
ECOG PS	0	131 (43)	76 (49)	19 (37)	22 (47)	9 (25)	5 (28)
	1	139 (45)	64 (42)	24 (46)	20 (43)	22 (61)	9 (50)
	2	16 (5)	2 (1)	6 (12)	4 (9)	3 (8)	1 (6)
	3	1 (0)	1 (1)	0 (0)	0 (0)	0 (0)	0 (0)
	Not performed	20 (7)	11 (7)	3 (6)	1 (2)	2 (6)	3 (17)
Sex	Male	152 (50)	67 (44)	26 (50)	26 (55)	22 (61)	11 (61)
	Female	155 (50)	87 (57)	26 (50)	21 (45)	14 (39)	7 (39)
Ann Arbor stage (*n* = 260)^b^	I	12 (5)	10 (7)	1 (2)		1 (3)	0 (0)
	II	39 (15)	21 (14)	11 (21)		6 (17)	1 (6)
	III	70 (27)	51 (33)	8 (15)		7 (19)	4 (22)
	IV	130 (50)	69 (45)	29 (56)		20 (56)	12 (67)
	Unknown	9 (3)	3 (2)	3 (6)		2 (6)	1 (6)
B symptoms	Yes	73 (24)	29 (19)	15 (29)	9 (19)	12 (33)	8 (44)
	No	219 (71)	122 (79)	34 (65)	36 (77)	19 (53)	8 (44)
	Unknown	15 (5)	3 (2)	3 (6)	2 (4)	5 (14)	2 (11)

*SD* standard deviation, *PS* performance status

^a^Other lymphoma entities comprised lymphocytic lymphoma (*n* = 8), not otherwise specified indolent NHL (*n* = 6), lymphoplasmocytoid lymphoma (*n* = 2), hairy cell leukemia (*n* = 1), and follicular lymphoma 3a (*n* = 1)

^b^Excluding immunocytoma

**Table 2:** Prognostic information at baseline

Characteristic		All *N* = 307
		*N* (%)
Bone marrow involved	Yes	121 (39)
Extranodal disease	Yes	186 (61)
	No	103 (34)
	Unknown	18 (6)
LDH > ULN	Yes	109 (36)
	No	182 (59)
	Unknown	16 (5)
Hemoglobin <12 g/dl	Yes	119 (39)
	No	187 (61)
	Unknown	1 (0)
IPI	≥1 risk factor	300 (98)
	≥2 risk factors	227 (74)
	≥3 risk factors	107 (35)
FLIPI only follicular lymphoma (*n* = 154)	Low risk	37 (24)
	Intermediate risk	59 (38)
	High risk	58 (38)

*LDH* lactate dehydrogenase in serum, *ULN* upper normal limit, *IPI* International Prognostic Index, *FLIPI* follicular lymphoma international prognostic index

One hundred and ninety-two (63 %) patients had at least one relevant concomitant disease. Most frequent diseases were hypertension (34 %), other tumor diseases (10 %), diabetes mellitus (9 %), and chronic lung disease (5 %).

### Treatment

Overall median time to treatment from primary diagnosis was 8.6 weeks and 9.9 in FL, 10.1 in MZL, 5.4 in IC, 5.5 in MCL, and 6.3 weeks in other entities, respectively. However, 25 % of the patients started treatment more than 54 weeks after primary diagnosis. The combination of bendamustine and rituximab (BR) was the most common regimen and administered to 289 (94 %) patients. In 33 (11 %) of these patients, BR was supplemented by dexamethasone or prednisone. Fourteen (5 %) patients received bendamustine monotherapy and four patients other combinations.

Regarding treatment regimens and lymphoma types, BR regimen without complementing steroids (*n* = 256) was more frequently used in FL compared to the other lymphoma entities (88 vs. 78 %, *p* = 0.02187).

Treatment was mostly administered at 4-week intervals. The vast majority of patients received bendamustine on days 1 and 2 of a treatment cycle (268 pts, 87 %). The median dose of bendamustine was 177 mg/m^2^ per cycle corresponding to 88.4 mg/m^2^ on days 1 and 2. Bendamustine doses were evenly distributed over the first 6 cycles. Median dose of bendamustine monotherapy was 133 mg/m^2^. At least one dose modification of bendamustine was documented in 79 (26 %) patients, a treatment delay (also) in 79 (26 %) patients. The occurrence of side effects was specified as the most frequent reason for a treatment delay (51 %).

The median dose intensity of bendamustine was 165 mg/m^2^ per 4 weeks for all treatment regimens overall, 166 mg/m^2^ per 4 weeks for BR on its own, and 132 mg/m^2^ per 4 weeks for bendamustine monotherapy. Median relative dose intensity was 0.9 for BR i.e., the proportion of the actual dose intensity in relation to the dose intensity as recommended by consensus guidelines (assuming bendamustine at 90 mg/m^2^ on days 1 and 2 of a 4-0week cycle) [5, 16]. The median number of bendamustine-based therapy was 6 cycles and did not differ by lymphoma type. In contrast, the mean number of cycles was 5.3 (+/− 1.5) for all, 5.5 (±1.4) for FL, 5.3 (±1.3) for MZL, 4.8 (±1.7) for IC, 5.1 (±1.6) for MCL, and 5.8 (±0.6) for others.

Maintenance therapy with rituximab following termination of bendamustine-based therapy was planned for 104 (33.9 %) patients and more frequently for patients with FL (81 of 142, 53 %; *p* = 0.0001).

### Best response

Complete response (CR) was documented as best response in 120 (43 %) patients and partial response in 121 (43 %) patients, corresponding to an overall response rate (ORR) of 86 % (95 % confidence interval 81–90, analysis set response rate *n* = 281). For 17 patients (6 %), best response was reported as stable disease, resulting in a disease control rate of 92 % (95 % CI 88–95). Ten patients (4 %) did not respond to treatment. Median and mean time to best response was 4.5 months overall (*n* = 240) and was comparable across lymphoma entities. ORR was 90 % in FL, 85 % in MZL, 78 % in IC, and 76 % in MCL. An overview of best response overall and by lymphoma type is displayed in Table 3.

**Table 3:** Best response overall and by lymphoma entity

Entity	CR *N* (%)	PR *N* (%)	SD *N* (%)	PD *N* (%)	Not evaluable *N* (%)	ORR *N* (%)	DCR *N* (%)
All entities (*n* = 281)	120 (43)	121 (43)	17 (6)	10 (4)	13 (5)	241 (86)	258 (92)
Follicular lymphoma (*n* = 142)	67 (47)	61 (43)	5 (4)	5 (4)	4 (3)	128 (90)	133 (94)
Marginal zone lymphoma (*n* = 46)	21 (46)	18 (39)	4 (9)	0 (0)	3 (7)	39 (85)	43 (94)
Immunocytoma (*n* = 45)	14 (31)	21 (47)	5 (11)	1 (2)	4 (9)	35 (78)	40 (89)
Mantle cell lymphoma (*n* = 33)	10 (30)	15 (46)	2 (6)	4 (12)	2 (6)	25 (76)	27 (82)
Other entities (*n* = 15)	8 (53)	6 (40)	1 (7)	0 (0)	0 (0)	14 (93)	15 (100)

*CR* complete response, *PR* partial response, *SD* stable disease, *PD* progressive disease, *ORR* overall response rate, *DCR* disease control rate (CR + PR + SD)

Interestingly, 13 of those 14 patients (ORR = 93 %) under bendamustine monotherapy showed a response. BR (*n* = 263) resulted in an ORR of 85 %. The ORR for the BR regimen without supplementation (*n* = 230) was 86 % compared to 79 % of the steroid supplemented BR regimen (*n* = 33).

ORR was determined to be influenced by the variable “age at therapy onset” (*p* = 0.0117; odds ratio = 0.9414) using a logistic regression model.

For evaluation of response, most of all, computed tomography (CT) was used (in 60 % of patients), followed by laboratory assessments (51 %), sonography (47 %), clinical examination (43 %), and bone marrow biopsy (10 %) (each method used at least once per patient during the observation period, multiple answers possible).

### Safety

A total of 429 bendamustine-related side effects of any grade were recorded. The majority of side effects resolved, and only 15 % persisted until the end of the observation period. Overall, 161 (50 %) of 323 patients (safety population) experienced at least one side effect. The most frequently reported CTCAE categories were blood/bone marrow (35 %), gastrointestinal (13 %), constitutional symptoms (8 %), dermatology/skin (5 %), neurology (3 %), infections (2 %), allergy/immunology, and pulmonary/upper respiratory (1 % of patients, respectively).

Fifty-four (17 %) patients experienced side effects of grade 3 (*n* = 47, 15 %) or grade 4 (*n* = 7, 2 %). Most frequently reported CTCAE categories for grade 3/4 events were blood/bone marrow (*n* = 43, 13 %), followed by infections (*n* = 5, 2 %), constitutional symptoms (*n* = 3, 1 %), and gastrointestinal (*n* = 2, 1 %) as well as cardiac arrhythmia, metabolic/laboratory, and musculoskeletal/soft tissue for one patient each, respectively.

Two grade 5 toxicities were documented. One death was associated with thrombocytopenia. The reason of death in the other case was unknown. Overall, there were 71 hospitalizations documented. Of those, 11 (15 %) were reported to be therapy-induced (by any of the agents administered), 12 (17 %) caused by the underlying malignant disease, and 10 (14 %) due to planned (elective) therapeutic or diagnostic interventions. Other frequently reported reasons for hospital admission were cardiac disease (*n* = 11, 29 %), infections (*n* = 10, 26 %), and gastrointestinal disease (*n* = 5, 13 %) which did not necessarily have to be related to lymphoma treatment.

Eleven serious adverse drug reactions for bendamustine were documented for ten patients according to the following CTCAE categories: constitutional symptoms (3), infection (3), blood/bone marrow (2), cardiac arrhythmia (1), gastrointestinal disorders (1), and death (not categorized, 1).

With regard to the safety set, 15 cases of death were documented during the observation period, 4 of these concerning patients with CLL. Reason for death was documented to be the underlying tumor disease for six patients and concomitant disease for two patients. Suspected relationship to therapy with bendamustine and/or rituximab was documented for one patient each. For four patients, cause of death was documented “due to other causes” and for one patient “cause of death unknown”.

## Discussion

In awareness of the inherent limitations of cross-trial comparisons, in particular between non-interventional and controlled interventional clinical trials, we aimed to evaluate if the effectiveness and safety of bendamustine as first-line treatment for indolent and mantle cell lymphomas as reported from clinical trials are representative for its use in routine clinical practice.

In clinical routine, bendamustine was predominantly given in combination with rituximab and at a median dose of 88 mg/m^2^ on days 1 and 2 at 4-week intervals which is in line with current consensus guidelines [16] and corresponds to the BR regimen evaluated in the first-line phase III trial reported by Rummel et al. [13]. Compared to the population of this relevant phase III trial, our patients were older (median age 71 vs. 64 years), less patients presented with stage IV disease (53 vs. 77 %), and fewer patients had B symptoms, bone marrow involvement, or extranodal disease. The proportion of the different lymphoma types was comparable with the exception of a somewhat lower proportion of patients with MCL. First-line bendamustine-based therapy in clinical routine practice resulted in a similar ORR (86 %) and CR (43 %) compared to Rummel et al. (93 and 40 %, respectively) [13], and patients with all lymphoma types had a similar benefit from treatment. Of note, there was no independent review in our trial which may have led to an overestimation of response. The limited follow-up of our study precludes further comparisons regarding progression-free survival or overall survival.

The reported side effects reflect the known safety profile of bendamustine. Overall, toxicity and, in particular, hematotoxicity reported in our trial was markedly lower than what has been reported for BR in first-line [13] or even from B or BR in rituximab-refractory NHL [8–12]. This probably has to be attributed to the non-interventional character of our study and a significant amount of underreporting of side effects that did not result in any therapeutic intervention. The short follow-up of our study does not allow an assessment of long-term toxicity. Yet, we think it is justified to state that in our study, first-line therapy of patients with indolent NHL or mantle cell lymphoma with bendamustine was manageable and well tolerated.

In conclusion, effectiveness and tolerability of bendamustine-based first-line therapy in patients with indolent lymphoma and mantle cell lymphoma were assessed as favorable in this non-interventional trial and were at least comparable to reported outcomes of recent literature. Our data are in alignment with the relevant treatment guidelines and recognize bendamustine-based therapy as a valid and valuable standard of care for patients with indolent lymphoma and mantle cell lymphoma in routine clinical practice.

### Integrity of research and reporting

The non-interventional study was approved by an appropriate ethics committee. All patients had to provide signed informed consent before their inclusion in the study.
